# Understanding antimicrobial resistance education among medical and veterinary students in Norway: a cross-sectional survey

**DOI:** 10.1093/jacamr/dlaf211

**Published:** 2025-11-20

**Authors:** Avis A Nowbuth, Aslak I Steinsbekk, Yngvild Wasteson, Ann-Katrin Llarena, Vikram S Parmar

**Affiliations:** Department of Neuromedicine and Movement Sciences, Faculty of Medicine and Health Sciences, Norwegian University of Science and Technology, Trondheim, Norway; Department of Research, Pan-African Organization for Health Education and Research (POHER), Manchester, MO, USA; Department of Public Health and Nursing, Faculty of Medicine and Health Sciences, Norwegian University of Science and Technology, Trondheim, Norway; Department of Paraclinical Sciences, Faculty of Veterinary Medicine, Norwegian University of Life Sciences, Ås, Norway; Department of Paraclinical Sciences, Faculty of Veterinary Medicine, Norwegian University of Life Sciences, Ås, Norway; Department of Design, Faculty of Architecture and Design, Norwegian University of Science and Technology, Trondheim, Norway

## Abstract

**Background:**

Antimicrobial resistance (AMR) poses a global health threat requiring effective education for future prescribers. Despite its urgency, there is limited evidence about how future prescribers are educated to mitigate AMR.

**Objectives:**

To investigate associations between AMR knowledge and (i) preferred teaching methods, (ii) self-reported competency, and (iii) recall of AMR-related curricular content among Norwegian medical and veterinary students.

**Methods:**

A cross-sectional survey of 110 students (61 medical, 49 veterinary) from two Norwegian universities assessed knowledge, preferred teaching methods, self-reported competency and self-reported curricular coverage. Bivariate analyses and multivariable linear regression identified associations with knowledge.

**Results:**

Formal lectures, guidelines, lab-based teaching and bedside teaching were the most preferred learning methods. Students reported that lab-based teaching had contributed to their learning about AMR, scoring higher on AMR knowledge (β = 0.89, *P* = 0.02), whereas those confident in knowing when to transition from IV to oral antibiotics had lower knowledge scores (β = −0.86, *P* = 0.031). Students reported lowest competency in antifungal-related topics.

**Conclusions:**

The findings highlight a disconnect, where confidence does not equate to knowledge, and exposure to the curriculum alone is insufficient to ensure clinical competence. AMR knowledge is associated with perceptions about lab-based learning, and misplaced confidence in specific skills. Antifungal-related topics require more attention in curricula.

## Introduction

Antimicrobial resistance (AMR) is a significant and growing public health threat globally, which impacts the health of humans and animals, agriculture, food security and environmental sustainability.^1,2^ Various studies highlight that knowledge and attitudes regarding AMR among healthcare providers are directly influenced by their training.^3–5^ However, the effectiveness of AMR education often varies due to differences in educational approaches, institutional priorities and students’ learning preferences.^4–7^ Thus, it is not surprising that the first objective of the WHO Global Action Plan on AMR emphasizes the need to ensure proper understanding and awareness among professionals.^2^ This is to be achieved by making AMR a core component in training and education.^2^ National policies reflect this; e.g. Norway’s ‘National One Health Strategy Against Antimicrobial Resistance 2024–2033’ states that the government will work to strengthen knowledge about AMR, One Health, and infection prevention and control in relevant graduate and postgraduate education.^8^

A systematic review of global studies suggested that gaps in knowledge and skills among healthcare students could be attributed to varied AMR education and shortcomings in undergraduate education.^9^ When asked about the overall usefulness of their education on antibiotic use, a significant portion of students rated it as useful,^7,10–12^ although there was significant variation between different teaching sites.^11,12^ A cross-sectional survey in 29 European countries found variability in knowledge, practical skills and attitudes towards antimicrobial stewardship (AMS) and AMR among medical students.^13–15^ On average, 66.1% of medical students expressed a desire for additional education on the use of antibiotics. Another scoping review highlighted the lack of data on knowledge for veterinary disciplines, reporting that 66.8% needed further education on prescribing, and 87.0% needed further education on AMR.^16^

Teaching methodologies influence student outcomes, retention and their perceived clinical decision-making skills.^9,10,17^ Traditional lecture-based approaches may not always meet the needs of diverse learners, with many students expressing preferences for interactive and practical educational methods.^4,14,18,19^

Importantly, learning preferences, defined as individual tendencies toward certain learning styles or methods, play an influential role in academic success and knowledge retention.^20,21^ By aligning educational content with students’ preferred learning modalities, educators can adopt a more effective and engaging learning experience.^22^ However, there remains limited understanding regarding how students’ preferred teaching methods and self-reported competencies are associated with their knowledge of AMR.

As prescribers and frontline responders to infection management, medical and veterinary professionals play key roles in implementing effective AMS strategies. As such, AMR education within medical and veterinary curricula is essential for equipping future professionals with the skills and knowledge required to curb the spread of resistant pathogens. Theoretical models of learning such as constructivist and schema theory emphasize how instructional methods can influence the depth, transferability and long-term retention of knowledge, and activate or build upon existing knowledge structures.^23–25^ Thus, it is especially important to learn more about how preferences are associated with knowledge among medical and veterinary students.

The aim of this study was therefore to investigate the association between knowledge of AMR and preferred teaching methods, self-reported competency and recall of previously taught AMR-related content among Norwegian medical and veterinary students.

## Methods

### Study design

A cross-sectional web-based survey using the Nettskjema, a secure online data collection platform provided by the University of Oslo, Norway (www.nettskjema.no), was performed. The Strengthening the Reporting of Observational Studies in Epidemiology (STROBE) guidelines were consulted in reporting the study.^26^

### Setting

The study was conducted at the Norwegian University of Science and Technology (NTNU) and the Norwegian University of Life Sciences (NMBU). NTNU is one of the four medical universities in Norway, and NMBU is the only university for veterinary education.

AMR education is integrated into two courses at NTNU: in second year (microbiology course) and in third year (medical expertise). In microbiology, students acquire foundational knowledge on microbial sensitivity to antimicrobial agents, resistance mechanisms, testing principles, and societal consequences of AMR.^27^ During their clinical year, their training includes clinical applications, emphasizing the role of resistant pathogens in organ-system diseases, diagnostics and therapeutic challenges.^28^

In the veterinary study programme at NMBU, the students are first exposed to AMR and AMS education in the second year in their veterinary microbiology and pharmacology courses, followed with practical application in the following years of their education.^29^ During the clinical rotations in the university clinics and field work, AMR and AMS questions are addressed related to disease management of individual patients and at herd levels in different species, including the zoonotic perspectives of AMR in a One Health perspective.

### Participants

Inclusion criteria were being a medical or veterinary student who was (i) registered at NMBU or NTNU, (ii) in their final year of study, and (iii) provided consent to complete the survey. Participants were recruited through direct emails to students from the class representative, physical campus advertisements, announcements in the learning management system, and Facebook class groups.

### Data collection

Data collection took place from 2 May 2024 to 10 September 2024, and again in February 2025 to increase the number of participants among veterinary students. The students were provided with a QR code and link to the online questionnaire.

### Questionnaire

A questionnaire was developed based on similar studies conducted by other universities across varied geographical locations.^7,10–12,15,30^ The survey was adapted for a Norwegian setting, and piloted among seven medical students and infectious disease and global health specialists, for readability, relevance and length. The final questionnaire included five areas: (i) demographics; (ii) knowledge test; (iii) teaching methods used; (iv) self-reported competency in AMR topics; and (v) self-reported recall of AMR curriculum coverage. All questions were in English.

### Dependent variable

AMR knowledge was assessed using eight clinical scenarios adapted from previous studies,^7,10–12,15,30^ and modified for a Norwegian setting in collaboration with veterinary specialists from NMBU and specialists from the Pan-African Organization for Health, Education and Research (POHER). Each scenario included a multiple-choice question with one correct answer, which was dichotomized as correct (1) or incorrect (0). The total AMR knowledge score was calculated as the sum of correct responses (range 0–8). The internal reliability of the clinical scenario items was 0.68, demonstrating moderate internal consistency.^31,32^

### Independent variables

There were four independent variables: (i) self-reported recall of AMR curriculum coverage; (ii) self-reported competency in AMR topics; (iii) teaching methods used to learn AMR; and (iv) demographics.

*Self-reported recall of AMR curriculum coverage* was used to assess how well the students thought that comprehensively specific AMR topics were covered. It consisted of one question ‘How well have the following topics been covered in your curriculum?’ with the 12 AMR topics. These questions were chosen to reflect the global AMR competency frameworks (WHO guidelines^33^) and reviewed by teachers to ensure that they aligned with the topics in the curriculum. The answering options for each topic were ‘poor’, ‘fair’, ‘good’, ‘very good’ and ‘excellent’, scored from 1 to 5. The answers were dichotomized into two categories: ‘good/very good/excellent’ (1) and ‘poor/fair’ (0). The internal reliability of the self-reported recall items was 0.90, demonstrating excellent internal consistency.^31,32^

*Self-reported competency in the AMR curriculum* was used to evaluate students’ perceptions on the same topics as described under *Self-reported recall of AMR curriculum coverage*, using the question ‘What is your competency level in the following areas?’ with the same answering options and categorization of the answers as above. The internal reliability of the competency items was 0.91, demonstrating excellent internal consistency.^31,32^

*Teaching methods* was used to evaluate the student’s perception of how well different educational methods helped them learn about AMR, using the question ‘How well have the following teaching methods contributed to your learning about AMR?’ The methods were selected based on literature and input from teachers. There were nine teaching methods listed: bedside teaching, traditional lectures, lab-based teaching, local handbook (guidelines), prescribed textbooks, applications for smart phones, computer-based tutorials, websites and learning games. The answering options and categorization of the answers were the same as above. In addition, a ‘not applicable’ option was included and used to identify how common the different teaching methods were. The internal reliability of the teaching method items was 0.68, demonstrating moderate internal consistency.^31,32^

*Demographics* included age, gender, and prior experience or exposure to AMR through work. Age was categorized into two groups: ≤25 years and ≥26 years. Gender was self-reported as female, male or prefer not to say, and collapsed into female and not female.

### Sample size

The sample size was calculated using a two-sample *t*-test formula to have power to show a difference corresponding to an effect size of 0.5 (moderate difference) between two sub-groups, with a significance level α = 0.05, a power (B) of 80, and an SD assumed equal between groups. We needed 64 students per group, and aimed for 130 respondents in total.

### Data analysis

Statistical analyses were performed using STATA/MP18. The responses are presented descriptively using frequencies, means, medians, SD and percentages. Chi-square and *t*-tests were used for bivariate comparison of knowledge scores and other variables. Effect sizes were calculated using Cohen’s *d*.^34^ Internal consistency of the multi-item scales were assessed using Cronbach’s alpha.^31,32^ The variables that had significant associations (*P* < 0.05) in the bivariate analyses were entered into a multivariable linear regression model to identify independent associations between knowledge scores and the other variables. Results are reported with 95% CI. Model assumptions were verified using residual plots and variance inflation factors (VIF < 5). Missing data were addressed by excluding missing responses, as missingness was <5% and assumed random.

### Ethics

The study was approved by the Norwegian Agency for Shared Services in Education and Research (SIKT) (Ref No.: 320862). Informed consent was obtained prior to participation and described on the primary page of the questionnaire. The study was voluntary, and the questionnaire posed no risk to the participants.

## Results

### Student population

A total of 237 medical and veterinary students who were enrolled in their programmes at the time of the study were invited to participate, with 110 completing the survey (response rate: 46%) and included in the analysis. Participants had a mean age of 26 (SD 2.06, range 24–30) years, with 60% identifying as female (Table 1). Forty-nine (45%) were veterinary students and 61 (55%) medical students. Prior AMR experience was reported by 68% (*n* = 75).

**Table 1.** Frequency of demographic characteristics and perception of teaching methods as good or poor in learning about AMR, with bivariable analysis of each variable with AMR knowledge scores

Variables		*n* (%)	Knowledge score Mean (SD)	Mean difference [95% CI]	*P* value	Cohen’s *d*
Female^a^	No	42 (40)	4.9 (1.3)	−0.35 [−0.87 to 0.18]	0.19	0.26
	Yes	64 (60)	5.2 (1.4)			
Age group	<25	48 (44)	5.3 (1.3)	0.38 [−0.13 to 0.90]	0.14	0.67
	>26	62 (56)	4.9 (1.4)			
Education	Medical	61 (55)	4.7 (1.4)	0.85 [0.37 to 1.34]	<0.001*	0.28
	Veterinary	49 (45)	5.6 (1.3)			
Work experience	Yes	75 (68)	4.9 (1.4)	0.36 [−0.18 to 0.91]	0.18	0.27
	No	35 (32)	5.3 (1.3)			
***Teaching methods***						
Formal lectures teaching	Good	105 (95)	5.0 (1.3)	−0.25 [−1.48 to 0.98]	0.69	0.18
	Poor	5 (5)	4.8 (1.5)			
Bedside teaching	Good	73 (68)	4.9 (1.3)	0.33 [−0.22 to 0.87]	0.23	0.45
	Poor	35 (32)	5.3 (1.3)			
Lab-based teaching	Good	67 (69)	5.2 (1.3)	−0.61 [−1.19 to −0.25]	0.04*	0.24
	Poor	30 (31)	4.6 (1.4)			
Local handbook (guidelines)	Good	77 (71)	5.1 (1.3)	−0.15 [−0.72 to 0.42]	0.61	0.11
	Poor	31 (29)	4.9 (1.4)			
Prescribed textbooks	Good	43 (40)	5.2 (1.2)	−0.30 [−0.83 to 0.24]	0.27	0.22
	Poor	64 (60)	4.9 (1.5)			
Applications for smart phones	Good	39 (58)	5.3 (1.2)	−0.05 [−0.61 to 0.52]	0.87	0.04
	Poor	23 (42)	5.3 (1.1)			
Computer-based tutorials	Good	29 (39)	5.5 (1.2)	−0.79 [−1.40 to −0.18]	0.011*	0.61
	Poor	46 (61)	4.7 (1.3)			
Websites	Good	49 (52)	5.4 (1.06)	−0.58 [−1.12 to −0.05]	0.03*	0.44
	Poor	46 (48)	4.8 (1.54)			
Learning games	Good	3 (19)	6 (1.7)	−0.77 [−2.58 to 1.04]	0.38	0.58
	Poor	13 (81)	5.2 (1.2)			

*P < 0. 05. Key: good = good/very good/excellent; poor = poor/fair.

^a^Collapsed into ‘female’ and ‘not female’ due to few responses in additional categories.

### Knowledge score

The mean AMR knowledge score, which could range from 0 to 8, was 5.0 (SD 1.35), with 70% (*n* = 77) scoring ≥5. The veterinary students scored higher than the medical students (mean difference 0.85, 95% CI 0.37–1.34) The question with the highest score concerned the principles of infection control and treatment with 94% correct, whereas managing a fungal outbreak in a multi-species setting received the lowest score with 30% correct (Table 2).

**Table 2.** Percentage of correct responses by topic, by discipline and overall

Clinical case scenario	Veterinary: % of *n* = 49	Medical: % of *n* = 61	Total: % of *n* = 110
1. Prevention control of methicillin-resistant *S. aureus* (MRSA) and *S. pseudintermedius* (MRSP)	90	80	85
2. Infection with ESBL-producing *E. coli*	43	31	36
3. Infection with resistant *Salmonella* spp.	92	75	83
4. *C. difficile* infection in a hospital and veterinary clinic	94	93	94
5. Antifungal resistance in a case of candidiasis	33	39	34
6. Management of TB caused by MDR-TB	67	85	77
7. MRSA transmission between pets and owners	92	39	63
8. Management of a fungal infection in a multi-species veterinary clinic	41	21	30
**Total correct answers, mean (SD)**	**5.5 (1.14)**	**4.7 (1.39)**	**5.0 (1.35)**

### Teaching methods

The most frequently used teaching methods were formal lectures, which all students reported encountering (100%, *n* = 110), whereas the least encountered was learning games (*n* = 16, 15%) (frequency not shown directly in Table 1 but is calculated by looking at the proportion answering ‘not applicable’). Significantly higher knowledge scores in the bivariate analysis were associated with being a veterinary student, and perceptions that lab-based teaching, computer-based tutorials and websites gave a good learning outcome (Table 1).

### Self-reported competency of AMR topic

There were differences in which AMR topic students reported competence in. Over 80% reported having good/very good/excellent confidence in foundational skills, whereas only a small proportion felt confident in choosing antifungals (6.4%) and antifungal dosing (6.4%) (Table 3). The AMR topics that were associated with a significantly higher knowledge score in bivariate analysis were transitioning from IV to oral antibiotics, knowing when to start antifungals, and choosing the correct antifungal for specific infections (Table 3).

**Table 3.** Frequency of self-reported competency in curriculum areas covered, with bivariate analysis of each competency area with AMR knowledge scores

Variable		Self-reported competency of AMR topics				
		*n* (%)	Knowledge score Mean (SD)	Mean difference [95% CI]	*P* value	Cohen’s *d*
Making an accurate diagnosis of infection	Good	97 (89)	5.0 (1.4)	0.15 [−0.068 to 0.97]	0.73	0.11
	Poor	12 (11)	5.2 (1.4)			
Understanding the basic mechanisms of AMR	Good	96 (87)	5 (1.4)	0.29 [−0.48 to 1.05]	0.46	0.21
	Poor	14 (13)	5.3 (1.3)			
Knowing when to start antibiotics	Good	100 (92)	5.0 (1.3)	0.20 [−0.74 to 1.14]	0.67	0.15
	Poor	9 (8)	5.2 (1.6)			
Choosing the correct antibiotic for specific infection	Good	90 (82)	5.0 (1.3)	0.14 [−0.52 to 0.80]	0.68	0.10
	Poor	20 (18)	5.15 (1.4)			
Knowledge of dosing and duration of antibiotic therapy for specific infections	Good	77 (70)	4.9 (1.3)	0.34 [−0.22 to 0.89]	0.23	0.25
	Poor	33 (30)	5.3 (1.5)			
How to de-escalate to narrower spectrum antibiotics	Good	39 (35)	4.7 (1.4)	0.49 [−0.03 to 1.01]	0.06	0.37
	Poor	71 (65)	5.2 (1.3)			
How and when to transition from IV to oral antibiotics	Good	27 (25)	4.3 (1.4)	0.93 [0.36 to 1.49]	<0.001*	0.72
	Poor	83 (75)				
How to interpret antibiograms	Good	19 (18)	4.8 (1.4)	0.26 [−0.42 to 0.93]	0.45	0.19
	Poor	87 (82)	5.0 (1.3)			
Understanding the spectrum of activity of commonly used antibiotics	Good	74 (67)	5.2 (1.3)	−0.51 [−1.0 to 0.03]	0.06	0.38
	Poor	36 (33)	4.7 (1.5)			
Knowing when to start antifungals	Good	20 (18)	5.6 (1.1)	−0.69 [−1.33 to −0.04]	0.038*	0.52
	Poor	90 (82)	4.9 (1.4)			
Choosing the correct antifungal for specific infections	Good	7 (6)	6.0 (0.8)	−1.03 [−2.1 to 0.00]	0.05*	0.77
	Poor	103 (94)	5.0 (1.4)			
Knowledge of dosing and duration of antifungal therapy for specific infections	Good	7 (6)	5.9 (0.7)	−0.88 [−1.9 to 0.16]	0.095	0.66
	Poor	103 (94)	5.0 (1.4)			

*P < 0.05. Key: good = good/very good/excellent; poor = poor/fair.

### Self-reported recall of AMR topics

There were differences in which AMR topic students reported as having been covered in their curriculum, following the same main patterns as the topics they reported competency in. Over 80% reported that diagnosing infections (100%), starting antibiotics (93%), understanding the basic mechanisms of AMR (93%) and choosing the correct antibiotic for specific infections (85%) were covered in their curriculum to a good/very good/excellent extent, whereas choosing antifungals (9%) and antifungal dosing (6%) were seldom recalled in the same way (Table 4).

**Table 4.** Frequency of perceived adequacy in learning about AMR, with bivariate analysis of each competency area with AMR knowledge scores

Variable		Self-reported recall of AMR topics				
		*n* (%)	Knowledge score Mean (SD)	Mean difference [95% CI]	*P* value	Cohen’s *d*
Making an accurate diagnosis of infection	Good	105 (100)	5.0 (1.4)			
	Poor					
Understanding the basic mechanisms of AMR	Good	102 (93)	5.1 (1.3)	−0.85 [−1.82 to 0.12]	0.0865	0.64
	Poor	8 (7)	4.3 (1.8)			
Knowing when to start antibiotics	Good	102 (93)	5.0 (1.3)	0.5 [−0.48 to 1.48]	0.31	0.37
	Poor	8 (7)	5.5 (1.5)			
Choosing the correct antibiotic for specific infection	Good	93 (85)	5.1 (1.3)	−0.60 [−1.30 to 0.09]	0.09	0.45
	Poor	17 (15)	4.5 (1.5)			
Knowledge of dosing and duration of antibiotic therapy for specific infections	Good	86 (78)	5.1 (1.3)	−0.15 [−0.78 to 0.47]	0.62	0.11
	Poor	25 (22)	4.9 (1.6)			
How to de-escalate to narrower spectrum antibiotics	Good	46 (42)	5.2 (1.3)	−0.31 [−0.83 to 0.2]	0.23	0.23
	Poor	64 (58)	4.9 (1.4)			
How and when to transition from IV to oral antibiotics	Good	32 (29)	5.0 (1.3)	0.10 [−0.47 to 0.66]	0.74	0.07
	Poor	78 (71)	5.1 (1.4)			
How to interpret antibiograms	Good	28 (25)	4.9 (1.2)	0.14 [−0.44 to 0.73]	0.63	0.11
	Poor	82 (75)	5.1 (1.4)			
Understanding the spectrum of activity of commonly used antibiotics	Good	86 (78)	5.2 (1.2)	−0.58 [−1.19 to 0.03]	0.06	0.44
	Poor	24 (22)	4.6 (1.8)			
Knowing when to start antifungals	Good	21 (19)	5.4 (1.1)	−0.48 [−1.13 to 0.16]	0.14	0.36
	Poor	89 (81)	4.9 (1.4)			
Choosing the correct antifungal for specific infections	Good	10 (9)	5.9 (1.1)	−0.95 [−1.82 to 0.08]	0.03*	0.72
	Poor	100 (91)	5.0 (1.3)			
Knowledge of dosing and duration of antifungal therapy for specific infections	Good	7 (6)	6.0 (1.3)	−1.03 [−2.06 to 0.00]	0.05*	0.77
	Poor	103 (94)	5.0 (1.3)			

*P < 0.05. Key: good = good/very good/excellent; poor = poor/fair.

The AMR topics that were associated with a significantly higher knowledge score in bivariate analysis were perceiving the curriculum as having good coverage of choosing the correct antifungal for specific infections, and knowledge of dosing and duration of antifungal therapy for specific infections (Table 4).

### Multivariable analysis

The multivariable linear regression analysis included the variables from the bivariate analysis with a *P* value ≤0.05 (Table 5). When the constant was included in the model, the students who perceived lab-based teaching as making a ‘good/very good/excellent’ contribution to their learning of AMR scored 0.89 points higher (95% CI: 0.12–1.66; *P* = 0.024) than those who scored ‘poor’ or ‘fair’. Students who perceived having ‘good/very good/excellent’ competency in how and when to transition from IV to oral antibiotics scored 0.86 points lower (95% CI: −1.63 to −0.08; *P* = 0.031).

**Table 5.** Multilinear regression analysis

Variable		Coefficient	95% CI	*P* value
Teaching methods	Lab-based teaching	0.89	0.12 to 1.66	0.024*
	Websites	−0.19	−0.96 to 0.59	0.629
	Computer tutorials	0.39	−0.31 to 1.09	0.269
Self-reported confidence	IV-to-oral transition	−0.86	−1.63 to −0.08	0.031*
	Starting antifungals	0.58	−0.48 to 1.63	0.279
	Correct antifungal	1.1	−1.46 to 4.47	0.418
Topic area covered	Correct antibiotic	0.40	−1.07 to 1.88	0.584
	Antifungal dosing	−0.09	−3.07 to 2.90	0.954
Discipline	University affiliation	−0.09	−0.87 to 0.71	0.830
Constant		4.415	2.90 to 5.92	0.000

## Discussion

The main findings were that students who reported higher confidence in transitioning from IV to oral antibiotic therapy demonstrated a lower knowledge score, whereas students who rated lab-based teaching favourably demonstrated significantly higher AMR knowledge scores. The students reported AMR learning from a range of methods, with formal lectures, the use of guidelines, lab-based teaching and bedside teaching as having contributed most. The students reported very low confidence in when to start antifungals and in choosing the correct antifungal.

Amongst all students, formal lectures and guidelines were reported as the teaching methods that contributed most to their learning. Although lectures are seen as important for teaching the fundamental principles,^7,10,11,35^ other similar studies have found that they were among the least popular methods to specifically improve preparedness for antibiotic prescribing.^7,10,15,36^ Such teaching methods have also been perceived as ‘least useful’,^36^ due to lacking practical application necessary for professional practice.^37^ Similarly, Spanish students did not feel they had received sufficient training in the judicious use of antibiotics through formal lectures.^38^ Despite formal lectures being the most prevalent source of learning, this method was not associated with higher knowledge scores in the multivariable analysis in this study.

Few students reported having tried learning games, and this study did not capture the type or quality of games used. Recent systematic reviews have shown that game-based learning methods (e.g. card games, board games or digital simulations about antibiotic use) significantly improve knowledge retention and stewardship behaviours compared with traditional lectures.^18,39,40^ The low exposure to learning games in our study therefore suggests a missed opportunity, as they could supplement or partly replace conventional teaching.^18,39,40^

Perceiving that lab-based teaching was helpful for AMR learning was the only variable associated with higher knowledge scores, aligning with the study by Abbo *et al.*^11^ in which hands-on stewardship simulations improved knowledge retention.^41^ In AMR education, lab-based teaching often involves hands-on instruction in diagnostics, sample handling, microbial isolation, testing antibiotic susceptibility and quality control.^42^ A prior study noted that lab-based methods may foster deeper understanding by contextualizing theoretical knowledge through practical application.^13^ Although lab sessions allow students to visualize and practise concepts, which can deepen understanding and retention,^41^ there is a recognition of the importance of connecting the subject matter to improve learning within microbiology and pharmacology teaching.^43^ Previous studies highlighted that lab teaching focuses on microbiology rotations within the context of medical education; however, Spanish, South African and Zambian students identified that lab-based microbiology rotations were one of the ‘least useful teaching methods’.^7,10,38^ Additionally, challenges such as resource limitations can hinder the effectiveness of these programmes since they are expensive and labour intensive.^44^ In our study, lab-based teaching was considered to be a good method of learning about AMR-related topics. By ensuring students have opportunities to directly observe and experiment with principles of antimicrobial action and resistance in lab-based settings, this can translate into better preparedness for real-world practice.

The inverse relationship between self-reported IV-to-oral transition competency and knowledge scores points towards a mismatch between students’ self-perceptions of confidence or preparedness and their actual knowledge scores. A plausible explanation is that students may be overestimating their competence in parts of antimicrobial prescribing. This phenomenon is suggestive of the Dunning–Kruger effect, where less proficient individuals mistakenly rate their abilities more highly due to a lack of insight into their own knowledge deficits.^45,46^ This has important implications for medical and veterinary education: without explicit feedback mechanisms and structured assessments, students may be unaware of these deficits, potentially carrying misplaced confidence into clinical practice.^45,46^ In East Africa, a study showed that students had low scores in knowledge about antibiotic use in clinical scenarios despite reporting feeling generally prepared,^47^ and further suggested that overconfidence had been associated with overprescribing.^10,11,47^ Studies in European countries suggested that high levels of reported preparedness or confidence on certain topics may represent overconfidence, as misdiagnosis is a leading cause of unnecessary antibiotic usage,^13,15^ and suggested that educational programmes should address possible overconfidence in students.^15^ The feeling of preparedness to diagnose infections accurately while feeling unprepared for prescribing was seen as potentially overconfidence in skills, thereby contributing to poor or inappropriate prescribing.^9,30,37,47,48^ Curricula should pair confidence-building activities (e.g. simulated stewardship scenarios) with thorough assessment to ensure alignment between perceived and actual knowledge. As such, our findings argue for the formal integration of metacognitive training, which could include formative feedback, peer comparison exercises and reflective self-assessments.^46^

Students showed varying proficiency across different AMR case scenarios. Notably, the highest competency was observed in managing *Clostridioides difficile* infections, whereas the lowest competency was observed in managing antifungal resistance cases and MDR fungal outbreaks, and the selection of the correct antibiotic for a specific infection. The gaps in antifungal therapy and antibiotic selection knowledge, despite adequate overall curriculum coverage, might indicate curricular blind spots. These topics are complex and may require dedicated instructional time or case-based learning to address them. The WHO emphasizes the need for antifungal agents to combat AMR effectively,^2,49^ a topic that is understudied, especially since resistant fungal strains complicate treatment protocols in both human and veterinary populations.^50^ Studies indicate that many healthcare professionals express a desire for more education on antifungal prescribing and resistance.^9,11,50^ When it comes to antibiotic selection, accurate selection is crucial for effective treatment of infections, as inappropriate use can lead to treatment failures.^51^ Veterinary students also face similar challenges regarding antibiotic selection, as they must understand the implications of their prescribing practices on both animal health and public health.^52^

This study encompassed both human (medical) and animal (veterinary) health trainees, and although it observed similar patterns across the groups, there may be specific needs in each sector. Veterinary students scored higher than medical students on the knowledge test, with the biggest difference seen in case scenario 7: the management of MRSA transmission between pets and owners. A possible explanation for this could be because veterinary programmes often integrate AMR topics earlier and more comprehensively into clinical training,^53^ whereas medical curricula may prioritize acute care over stewardship.^49^ Veterinary programmes have increasingly emphasized the One Health approach due to the increase in zoonotic diseases, which poses significant risks to human and animal health.^54^ In the ESEVT system, one of the Day One Competences is to “understand and apply principles of One Health to ensure veterinary Good Clinical Practice, and research-based and evidence-based veterinary medicine.”^55^

Our findings advocate for integrating interactive, practical teaching methods into AMR curricula in Norway. Given the strong association between lab-based methods and higher knowledge scores, lab-based teaching should be prioritized to enhance competency in AMR management. Other active learning methods offer an opportunity to motivate and encourage students to learn more about AMR-related topics as an adjunct to traditional lectures. Additionally, the discrepancies identified between perceived and actual competencies highlight the importance of employing objective assessments alongside self-reported measures to improve the accuracy of competence evaluations in medical and veterinary education. Aligning curriculum design with frameworks like the WHO Competency Framework for Health Workers’ Education and Training on Antimicrobial Resistance (2018), which emphasizes interdisciplinary competency, could address these gaps.^56^

### Strengths and limitations

To our knowledge, this is the first study to compare AMR educational experience with objective knowledge scores in medical and veterinary student populations in Norway. The inclusion of a medical and a veterinary university allowed a view across disciplines. The knowledge test was based on previously used validated surveys.

However, there are some important limitations. The sample size achieved (*n* = 110) fell below the target a priori power calculation (*n* = 128) thereby decreasing the precision of the results. Although the study retained approximately 75% power to detect a medium effect size (*d* = 0.50), the reduced sample may have limited our ability to detect smaller but potentially meaningful effects. No data were available for those eligible but not participating, making it difficult to assess the sample’s representativeness of those invited. The sample is, however, similar to what is known generally about medical and veterinary students regarding gender and age.^57^ Still, data were collected from one medical school in Norway only, and findings may not generalize to other medical programmes with different teaching in AMR. The self-reported data may be subject to recall bias or social desirability bias,^58–60^ potentially skewing participants’ accounts of their educational experiences. The voluntary nature of the survey introduces the possibility of selection bias,^60,61^ as respondents likely overrepresented students with an interest in AMR or who had strong opinions about their education. The extended data collection period raises the possibility of temporal confounding, as later respondents had up to 3 months less exposure time than those who participated in 2024. Variations in students’ academic backgrounds, faculty teaching approaches and institutional priorities were not assessed. Additionally, there may be other unobserved factors such as resource availability, cultural attitudes towards learning, or additional external exposures, like campaigns or training opportunities, that could further shape outcomes.^60^ Finally, combining the answers from medical and veterinary students to have a One Health perspective, might have hidden differences between them.

This study provides insights into medical and veterinary students’ perceptions of AMR teaching and its association with knowledge. Lab work was the only factor linked to higher knowledge. The observed disconnect between confidence and competence highlights that education should prioritize proficiency in antimicrobial stewardship rather than simply boosting self-confidence.
