# Correction to: Understanding antimicrobial resistance education among medical and veterinary students in Norway: a cross-sectional survey

**DOI:** 10.1093/jacamr/dlaf232

**Published:** 2025-12-04

This is a correction to: Avis A Nowbuth, Aslak I Steinsbekk, Yngvild Wasteson, Ann-Katrin Llarena, Vikram S Parmar, Understanding antimicrobial resistance education among medical and veterinary students in Norway: a cross-sectional survey, *JAC-Antimicrobial Resistance*, Volume 7, Issue 6, December 2025, https://doi.org/10.1093/jacamr/dlaf211

The following changes have been made to the originally published manuscript. The title has been emended to read: “Understanding antimicrobial resistance education among medical and veterinary students in Norway: a cross-sectional survey” instead of: “Understanding antimicrobial resistance education among medical and?veterinary students in Norway: a cross-sectional survey”.

